# Hepatocyte-specific mitogen-activated protein kinase phosphatase 1 in sexual dimorphism and susceptibility to alcohol induced liver injury

**DOI:** 10.3389/fimmu.2024.1316228

**Published:** 2024-02-01

**Authors:** Mary Nancy Walter, Diego Montoya-Durango, Walter Rodriguez, Yali Wang, JingWen Zhang, Julia H. Chariker, Eric C. Rouchka, Claudio Maldonado, Anton Bennett, Craig James McClain, Shirish Barve, Leila Gobejishvili

**Affiliations:** ^1^ Department of Physiology, School of Medicine, University of Louisville, Louisville, KY, United States; ^2^ Department of Medicine, School of Medicine, University of Louisville, Louisville, KY, United States; ^3^ Department of Neuroscience Training, University of Louisville, Louisville, KY, United States; ^4^ Kentucky IDeA Networks of Biomedical Research Excellence, (KY INBRE) Bioinformatics Core, University of Louisville, Louisville, KY, United States; ^5^ Department of Biochemistry and Molecular Genetics, University of Louisville, Louisville, KY, United States; ^6^ Department of Pharmacology, Yale University School of Medicine, New Haven, CT, United States; ^7^ Robley Rex Veterans Affairs (VA) Medical Center, Louisville, KY, United States; ^8^ Department of Pharmacology and Toxicology, University of Louisville, Louisville, KY, United States; ^9^ Alcohol Research Center, University of Louisville, Louisville, KY, United States; ^10^ Hepatobiology and Toxicology Center, University of Louisville, Louisville, KY, United States

**Keywords:** MKP1, alcohol-associated liver disease, sexual dimorphism, inflammation, metabolism

## Abstract

**Background:**

It is well established that females are more susceptible to the toxic effects of alcohol, although the exact mechanisms are still poorly understood. Previous studies noted that alcohol reduces the expression of mitogen-activated protein kinase phosphatase 1 (MKP1), a negative regulator of mitogen-activated protein kinases (MAPK) in the liver. However, the role of hepatocyte- specific MKP1 in the pathogenesis of alcohol-associated liver disease (ALD) remains uncharacterized. This study aimed to evaluate the role of hepatocyte-specific MKP1 in the susceptibility and sexual dimorphism in alcohol-induced liver injury.

**Methods:**

C57Bl/6 mice were used in an intragastric ethanol feeding model of alcohol-associated steatohepatitis (ASH). Hepatocyte-specific *Mkp1^-/-^* knockout and (*Mkp1^+/+^* “f/f” male and female mice were subjected to the NIAAA chronic plus binge model. Primary mouse hepatocytes were used for *in vitro* studies. Liver RNA sequencing was performed on an Illumina NextSeq 500. Liver injury was evaluated by plasma alanine transaminase (ALT), hepatic ER stress and inflammation markers. Statistical analysis was carried out using ANOVA and the unpaired Student’s t-test.

**Results:**

ASH was associated with the severe injury accompanied by increased endoplasmic reticulum (ER) stress and significant downregulation of *Dusp1* mRNA expression. *In vitro*, ethanol treatment resulted in a time-dependent decrease in *Dusp1* mRNA and protein expression in primary hepatocytes in both males and females; however, this effect was significantly more pronounced in hepatocytes from females. *In vivo*, female mice developed more liver injury in a chronic plus binge model which was accompanied by a significant decrease in liver *Dusp1* mRNA expression. In comparison, liver *Dusp1* was not changed in male mice, while they developed milder injury to alcohol. *Mkp1* deletion in hepatocytes led to increased alcohol induced liver injury, ER stress and inflammation in both sexes.

**Conclusion:**

Hepatocyte Mkp1 plays a significant role in alcohol induced liver injury. Alcohol downregulates Mkp1 expression in hepatocytes in a sex dependent manner and could play a role in sexual dimorphism in increased female susceptibility to alcohol.

## Introduction

Alcohol-associated liver disease (ALD) is a significant health problem in the United States and worldwide (1–3). ALD is a multifactorial disease that manifests as alcohol-associated fatty liver, steatohepatitis, and fibrosis/cirrhosis (4). Patients with ALD and cirrhosis develop acute alcohol-associated hepatitis (AH), which has the highest mortality (5, 6). Despite extensive research and clinical trials, there is no FDA-approved therapy for any stage of ALD(7–9). Previous research has shown that c-Jun N-terminal kinase (JNK) plays a significant role in hepatocyte death (10–12). Specifically, sustained activation of JNK has been shown to be detrimental for hepatocyte survival (12, 13). Notably, the pathogenic role of prolonged JNK activation has been described in various liver pathologies including nonalcoholic and alcohol-associated liver diseases (12, 13). Due to its significant role in hepatocyte injury, it has been proposed that targeting the signaling pathways leading to prolonged JNK activation is an important strategy in treating acute and chronic liver disease (12, 13). In several *in vivo* models of ALD, increased and sustained activation of JNK has been demonstrated to contribute to liver injury (12, 14, 15). The crucial role of JNK1 and 2 in ALD has been confirmed by using pharmacological and genetic approaches in animal models (16, 17).

Mitogen-activated protein kinase phosphatase 1 (MKP1, gene name *Dusp1*), the archetypal member of the dual-specificity phosphatases, has been identified as a negative regulator of both JNK and p38 MAPK through dephosphorylation (18–20). Previous investigations have suggested that an ethanol-mediated increase in oxidative stress and proteasomal degradation of MKP1 protein leads to JNK-mediated apoptosis of hepatocytes after acute exposure to ethanol (21). Later studies by Cederbaum’s group suggested that decreased MKP1 protein levels in the liver contribute to sustained JNK activation and liver injury mediated by CYP2E1 and TNF toxicity (22). More recently, it was demonstrated that overexpression of MKP1 in the liver prevented alcohol-induced mitochondrial dysfunction, restored mitophagy, and attenuated liver injury (23). Clinically, RNA sequencing analysis of livers from healthy donors and patients with alcohol-associated hepatitis (AH) showed that *Dusp1* levels were significantly lower in patients with AH (24).

MKP1 is also a pivotal player in the control of innate immune response (25–27) and tissue inflammation (28). Inflammation plays a significant role in ALD (29). Gut-derived microbial products, pathogen-associated molecular patterns (PAMPs) and damage-associated molecular patterns (DAMPs) in ALD lead to the activation of the innate immune system via pattern recognition receptors (PRRs) (29). This leads to the release of various cytokines and chemokines, recruitment of neutrophils and monocytes to the liver, and tissue injury (29). Whole body *Mkp1* knockout mice have been extensively used in various models of infection and inflammation (30). However, dissecting tissue-specific roles for MKP1 is challenging due to its widespread expression in multiple organs and cells. Therefore, the use of cell type specific *Mkp1* knockout mice are necessary to understand the role of MKP1 in specific cellular responses. Hence, to identify the role of MKP1 in alcohol induced hepatocyte injury, we employed mice in which MKP1 is deleted only in hepatocytes. Our data demonstrate that alcohol-associated steatohepatitis is associated with significantly lower hepatic *Dusp1* mRNA levels. Deleting MKP1 in hepatocytes augments alcohol-induced liver injury in males and females. However, alcohol affects MKP1 expression differently in males and females, which might be a contributing factor to sexual dimorphism in susceptibility to ALD.

## Materials and methods

### Alcohol-associated steatohepatitis mouse model

Male C57Bl/6 mice were subjected to the ASH regimen, which consisted of feeding a solid Western diet high in cholesterol and saturated fat (HCFD) or regular chow *ad libitum* for two weeks. This initial acclimation period was followed by intragastric (ig) feeding of ethanol and a high-fat liquid diet (36%Cal corn oil) at 60% of their total caloric intake plus *ad libitum* intake of HCFD for the remaining 40% of calories. The ethanol dose was increased to 27 g/kg/day over an eight-week period, and pair-fed (PF) control mice were fed an isocaloric high-fat liquid diet. Additionally, mice were subjected weekly to an alcohol binge (5 g/kg) from the second week of ig feeding as described(31). After eight weeks of feeding, mice were euthanized between 10:30am to 1pm one day after the final binge. All experimental protocols were approved by the University of Southern California Institutional Animal Care and Use Committee.

### Chronic plus binge ALD model

Hepatocyte-specific *Mkp1* knockout mice were generated using a Cre-lox mechanism as described (32). Male and female hepatocyte-specific *Mkp1^-/-^* knockout (abbreviated as liver-specific knockout, LSKO) and their littermate control (*Mkp1^+/+^* “f/f”) mice were subjected to the chronic plus binge model (33). Mice were housed in a pathogen-free, temperature-controlled animal facility with 12 h light/12 h dark cycles. Mice were pair-fed the Lieber-DeCarli liquid diet containing either isocaloric maltose dextrin (PF) or 5% (w/v) ethanol (AF) *ad libitum* for ten 10 days. Food consumption was monitored daily. f/f and LSKO mice on alcohol diet consumed in average 8 ml/day. Pair-feeding was done by giving the same amount of control diet (8 ml) to mice in PF group. On day 11, mice on the alcohol-fed diet (AF) were given 31.5% (v/v) ethanol via oral gavage, while mice on the isocaloric maltose dextrin control diet (PF) were gavaged with water. Mice were euthanized 9 hours after gavage. There were 8-10 mice in each experimental group. Whole blood was collected, and plasma samples were stored at -80°C until processing. Tissues were snap-frozen and stored at -80°C before analysis. Additional tissues were fixed in 10% neutral-buffered formalin and cryo-preserved for immunohistochemical analysis. All experimental protocols were approved by the University of Louisville Institutional Animal Care and Use Committee in accordance with the National Institutes of Health Office of Laboratory Animal Welfare Guidelines.

### Isolation of mouse hepatocytes

Hepatocytes were isolated from male and female hepatocyte-specific *Mkp1* ^-/-^ knockout (LSKO) and their littermate control (“f/f”) mice by collagenase perfusion as described (34). Cells were plated in 6 well culture plates (cat# 92006, Midwest Scientific, Valley Park, MO) at 0.5x10^6^cells/well. After attachment, media was replaced with DMEM supplemented with 5% FBS and 1% P/S (cat# 15140-122, Gibco). Ethanol (50 mM) was added to ethanol-treated cells for indicated times.

### Cytokine measurement

Liver cytokines were measured using the MesoScale Discovery (MSD) platform. Total liver tissue samples were lysed with a buffer of 200 mM NaCl, 5 mM EDTA, 10 mM Tris, 10% glycerin, and protease/phosphatase inhibitors (cat#78445, ThermoFisher Scientific). The incubation of liver tissue lysates with the V-Plex MULTI-SPOT Assay System from MSD was carried out overnight at 4°C to increase sensitivity of the assay. Proinflammatory Panel 1 (mouse) and Cytokine Panel 1 (mouse) kits were used (MesoScaleDiscovery, Rockville, MD, cat # K15048D and K152245G, respectively). The plate was read with a MESO QuickPlex SQ 120 imager and analyzed using Discovery Workbench v4.0 software. The assay was performed according to the manufacturer’s instructions.

### Measurement of liver injury markers

Plasma alanine aminotransferase (ALT) and aspartate aminotransferase (AST) levels were measured using colorimetric enzymatic assay kits (cat# MAK052 (ALT) and cat# MAK055 (AST), Millipore Sigma, St. Louis, MO), according to the manufacturer’s instructions.

### Plasma glucose measurement

Plasma glucose levels were measured using glucose colorimetric detection kit (Invitrogen, Carlsbad, CA; Cat# EIAGLUC). Plasma was diluted 20 times before measurement. Data are presented in mg/dL.

### RNA isolation and reverse transcription-qPCR analysis

Total RNA from mouse liver tissue and cells was isolated using TRIzol Reagent (Invitrogen, Carlsbad, CA, USA). RT-qPCR was performed as described previously (35, 36). Specific primers were purchased from Integrated DNA Technologies (IDT, Coralville, IA, USA). Primer sequences are presented in **Table 1**.

**Table 1:** Primers for quantitative RT-PCR.

Mouse *Atf-3*	Forward	5’-GCGCTGGAGTCAGTTACCGTCA-3’3’
	Reverse	5’-TTCTTCAGGGGCCGCCTCAGAC-3’3’
Mouse *Atf-4*	Forward	5’-AAGCCATGGCGCTCTTCACGA-3’3’
	Reverse	5’-AGTCCCCCGCCAACACTTCG-3’3’
Mouse *Chop*	Forward	5’-CCGGAACCTGAGGAGAGAGTGTT-3’
	Reverse	5’-AGCTGCCATGACTGCACGTGG-3’
Mouse *Gadd34*	Forward	5’-AGAGAAGCCAGAATCACCTTG-3’
	Reverse	5’-GAAGTGTACCTTCCGAGCTTT-3’
Mouse *Dusp1*	Forward	5’-GCTATTGACTTCATAGACTCC-3’
	Reverse	5’-TCTGCTTCACAAACTCAAAG3’
Mouse *Gadd45a*	Forward	5’-AGATCCATTTCACCCTCATC-3’
	Reverse	5’-ATTGTGATGAATGTGGGTTC-3’
Mouse *Gadd45b*	Forward	5’-CCTGGTCACGAACTGTCATAC-3’
	Reverse	5’-GGTTATTGCCTCTGCTCTCTT-3’
Mouse *Pparα*	Forward	5’-CATCGAGTGTCGAATATGTGG-3’
	Reverse	5’-CGAATAGTTCGCCGAAAGAA-3’
Mouse *Ppargc1α*	Forward	5’-ACAGCTTTCTGGGTGGATTG-3’
	Reverse	5’-CGCTAGCAAGTTTGCCTCAT-3’
Mouse *Spp1*	Forward	5’-GGATGACTTTAAGCAAGAAACTCT-3’
	Reverse	5’-ACAGAATCCTCGCTCTCTGC-3’
Mouse *Dusp6*	Forward	5’-ATGATGAGGTCTTCAGTCTC-3’
	Reverse	5’-CAAATACCCCTTGAGACAC-3’
Mouse *Pklr*	Forward	5’-GTGAAGAAGTTTGATGAGATCC-3’
	Reverse	5’-CAAGAAAACCTTCTCTGCTG-3’
Mouse *G6pc*	Forward	5’-CCTATAATAAAGCAGTTCCCTG-3’
	Reverse	5’-ATCCATACGTTGGCTTTTTC-3’
Mouse *Gys2*	Forward	5’-CCGTGGTAGTGTTTTTCATC-3’
	Reverse	5’-TCTTCCCAAACTTCTCCTTC-3’

### Western blot analysis

Liver tissue was lysed using RIPA buffer (50mM Tris HCl at pH 8, 150mM NaCl, 1mM EDTA, 1% NP40, 0.5% Na-deoxycholate, and 0.1% SDS) containing protease and phosphatase inhibitors (ThermoFisher Scientific). Proteins (25 µg of cell lysate and 50 µg of liver tissue lysate) were analyzed by SDS-polyacrylamide gel electrophoresis using a BioRad electrophoresis system (Hercules, CA, USA). Membranes were stripped using Restore™ PLUS Stripping Buffer (cat# 46430, Thermo Scientific, Rockford, IL, USA) and reprobed using another antibody. Immunoreactive bands were visualized using enhanced chemiluminescence light detection reagents (cat# WBLUF0500, Millipore, Burlington, MA, USA). Quantification was performed with Image Lab™ software (Bio-Rad, version 5.2.1). MKP1 antibody was purchased from Santa Cruz Biotechnology (sc-373841) and used at 1:500 dilution. CYP2E1 antibody was from Abcam (ab28146, dilution 1:2500). Antibodies to GAPDH (#5174), phospho-JNK (Thr183/Tyr185, #9251), JNK (#9252), CHOP (#5554), ATF3 (#18665), ATF4 (#11815), phospho-Akt (Ser473) and Akt (#9272) were purchased from Cell Signaling Technology.

### TUNEL staining

5 µm of paraffin sections were de-paraffinized in xylene and rehydrated in a series of ethanol and water, then PBS. Sections were then stained with Chemicon^®^ ApopTag^®^ Peroxidase *In Situ* Apoptosis Detection Kit (Sigma-Aldrich, Burlington, MA) until equal staining was achieved across samples. The sections were then dehydrated and mounted.

### RNA sequencing analysis

Total RNA was extracted from liver tissue by TRIzol and a NanoDrop 2000 spectrophotometer (ThermoFisher) was used to determine RNA concentration. mRNA was enriched with magnetic beads containing oligo(dT), and Fragmentation Buffer was added to the mRNA to cut fragments into short fragments. Then, short fragments were used as templates to synthesize the first cDNA strand with random hexamers. Buffer, dNTPs, RNase H, and DNA polymerase I were added to synthesize the second cDNA strand. After purification (QiaQuick PCR kit, cat # 28104), cDNAs were repaired and were added with base A, then were recovered with the target size fragments by agarose gel electrophoresis. PCR amplification was conducted to complete the entire library preparation. HiSeq2000 (Illumina) was used for the sequencing of the constructed library. Quality control was performed using FastQC (v.0.10.1) (37). Sequenced reads were of good quality, and no sequence trimming was necessary. The sequences were aligned to the mouse reference assembly (mm10) using the TopHat2 aligner (v.2.0.13) (38) with alignment rates above 97.5%. Gene read counts were generated using Cuffnorm (39) with FPKM normalization. Differential expression analysis was performed using Cuffdiff2 (v.2.2.1) (39).

### Statistical analysis

Statistical analysis was performed using GraphPad Prism version 9 for Windows (GraphPad Software, Inc., La Jolla, CA). Data were analyzed, as appropriate, by the Student’s t-test (for two groups) or by two-way analysis of variance (ANOVA) with Bonferroni post-test analysis (for greater than two groups). Data points with a distance greater than two standard deviations from the mean were considered outliers and removed from the data set. Differences were considered statistically significant at p< 0.05.

## Results

### Hepatic *Mkp1* mRNA expression is significantly decreased in a mouse model of alcohol-associated steatohepatitis

**Figure 1:**
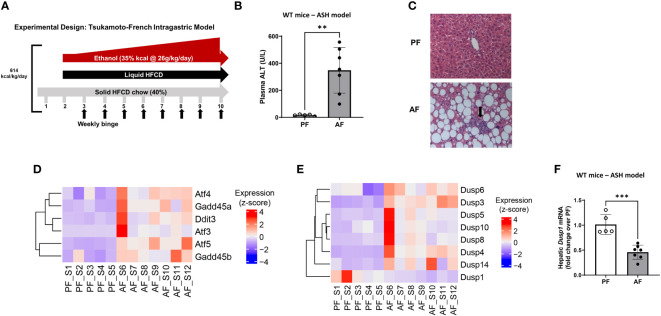
Intragastric chronic administration of ethanol and high caloric diet results in liver steatosis and altered expression of *Dusp* gene family. **(A)** Graphical overview of the murine alcohol-associated steatohepatitis (ASH) model. **(B)** Plasma ALT levels in pair-fed and alcohol-fed male C57Bl/6 mice in the ASH model. n=6 in PF, n=7 in AF groups, unpaired t-test, **P ≤ 0.01. **(C)** Hematoxylin-Eosin staining shows immune cell infiltration into the liver tissue of ASH mice (arrow) (10X magnification shown). **(D)** Heatmap showing increased ER stress in ASH mice, **(E)** Heatmap of significantly altered Dusps (MKPs). **(F)** Real-time qPCR analysis of total liver tissue from pair-fed (PF) and alcohol-fed (AF) mice showed downregulation of MKP1 (Dusp1) in alcohol-fed mice. Unpaired t-test, ***P ≤ 0.001.

Several murine models of alcohol exposure have been developed to mimic different stages of ALD to study its mechanisms (for review see (40)). Tsukamoto’s group developed intragastric chronic alcohol feeding model of ASH, which recapitulates several features of human ASH (steatosis, inflammation and pericellular fibrosis). Liver injury is more severe in this model, as indicated by high ALT levels with some mice reaching 500 U/L (31). In this model, a hybrid feeding involving 40% of total calories from solid chow, and the remaining intake in the form of an intragastric liquid diet was administered as shown in **Figure 1A**. As reported previously, this approach led to severe liver injury as demonstrated by high serum ALT levels, immune cell infiltration, and lipid accumulation in the liver (**Figures 1B, C**). Investigation of differential changes in gene expression through RNA sequencing analysis showed that alcohol-fed (AF) mice had significantly elevated levels of ER stress markers such as activating transcription factor 3 (ATF3) and 4 (ATF4), DNA damage-inducible transcript 3, also known as C/EBP homologous protein (CHOP), DNA damage-inducible alpha (GADD45a) and beta (GADD45b) compared to pair-fed counterparts (**Figure 1D**). RNA sequencing analysis also showed that alcohol feeding resulted in significant changes in the expression of several dual specificity phosphatases (Dusps or Mkps) (**Figure 1E**). Notably, while *Dusp3*, *Dusp4, Dusp5*, *Dusp6*, *Dusp8*, *Dusp10*, *and Dusp14* were upregulated, only *Dusp1 (Mkp1)* was significantly downregulated, which was confirmed to be significantly decreased as shown by real time qPCR analysis (**Figure 1F**). This result shows that exposure to alcohol led to decreased liver expression of *Dusp1* mRNA levels and increased ER stress-associated genes.

### Deletion of Mkp1 in hepatocytes augments alcohol-induced liver injury

**Figure 2:**
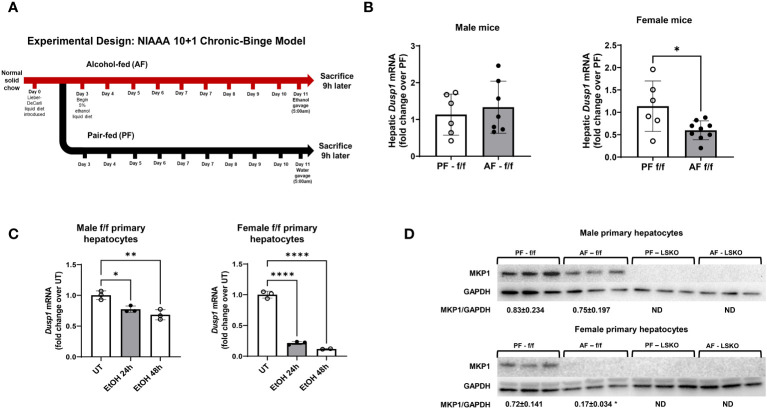
Females exhibited more significant Mkp1 downregulation than males in response to alcohol. **(A)** Chronic plus binge model of ALD, **(B)** Hepatic *Dusp1* (MKP1) mRNA in f/f/ male and female mice by RT-qPCR analysis. **(C)** *Dusp1* mRNA levels in male and female primary hepatocytes treated with 50 mM ethanol for 24 or 48 hours. **(D)** Representative image of Western Blot analysis of Mkp1 protein expression in male and female primary hepatocytes treated with 50 mM ethanol for 24 hours. Densitometric ratios to GAPDH (loading control) are shown. *P ≤ 0.05, **P ≤ 0.01, ****P ≤ 0.0001.

To show a causal role of hepatocyte MKP1 in alcohol-induced liver injury, we subjected wild type (f/f) and hepatocyte-specific *Mkp1* knockout (LSKO) male and female mice to a NIAAA chronic plus binge model of ALD (**Figure 2A**). Alcohol feeding did not alter hepatic *Mkp1* mRNA levels in male f/f mice, while alcohol-fed female f/f mice showed significantly lower levels when compared to pair-fed counterparts (**Figure 2B**). To examine the direct effect of alcohol on hepatocyte MKP1, we isolated hepatocytes from male and female mice and treated them with 50 mM ethanol for 24 and 48 hours. mRNA and protein expression analyses of MKP1 showed that ethanol had a much more pronounced effect on female hepatocytes (**Figures 2C, D**). Hepatocytes from LSKO mice showed no expression of MKP1 protein as expected (**Figure 2D**).

**Figure 3:**
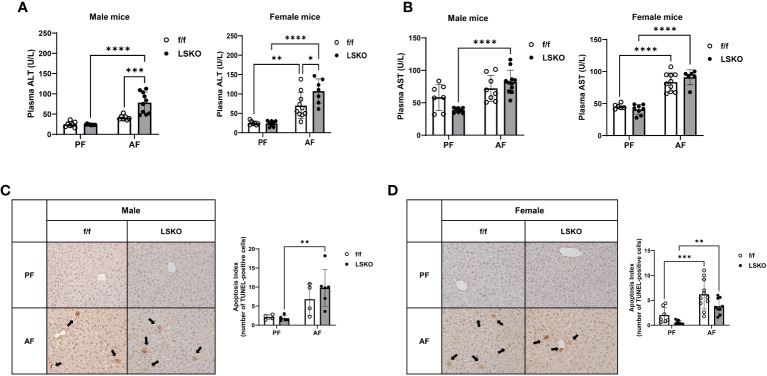
Deletion of Mkp1 in hepatocytes increases vulnerability to alcohol-induced liver injury. **(A)** Plasma ALT and **(B)** AST (U/L) from male and female mice showed increased liver injury in LSKO mice compared to their f/f counterparts in the chronic plus binge model. **(C, D)**. TUNEL staining of livers from male and female f/f and LSKO mice with quantification (20X magnification shown). Two-way ANOVA, **P ≤ 0.01, ***P ≤ 0.001, ****P ≤ 0.0001.

Alcohol feeding *in vivo* resulted in a modest increase in ALT levels in male f/f mice, while it led to a significantly higher levels of ALT in LSKO mice (**Figure 3A**). AST levels were also much higher in LSKO male mice (**Figure 3B**). In contrast, alcohol feeding of female f/f mice led to a significant increase in ALT levels, which was further elevated in LSKO mice (**Figure 3A**). AST levels were also increased in AF fed f/f and LSKO female mice, however there was no difference between the genotypes (**Figure 3B**).

To evaluate the degree of apoptosis in the livers, tissue samples were stained for terminal deoxynucleotidyl transferase dUTP nick end labeling (TUNEL) (**Figure 3C**). Five images were gathered randomly from each liver, and stained TUNEL-positive cells were counted (**Figure 3D**). A response similar to that of ALT levels was observed in apoptosis indices for each group, although there were some differences between male and female mice. Specifically, in LSKO-PF female mice, fewer apoptotic cells were found; nevertheless, alcohol increased apoptotic cell numbers similar to their f/f counterparts. Taken together, these observations show that Mkp1 deletion augmented alcohol induced liver injury in both males and females. However, alcohol had a more pronounced effect on liver injury and Mkp1 expression in f/f females, which suggests that alcohol mediated decrease in Mkp1 expression might be a contributing factor to their susceptibility to alcohol induced liver injury compared to males.

### Effect of sex and Mkp1 deletion on lipid metabolism in chronic plus binge model

**Figure 4:**
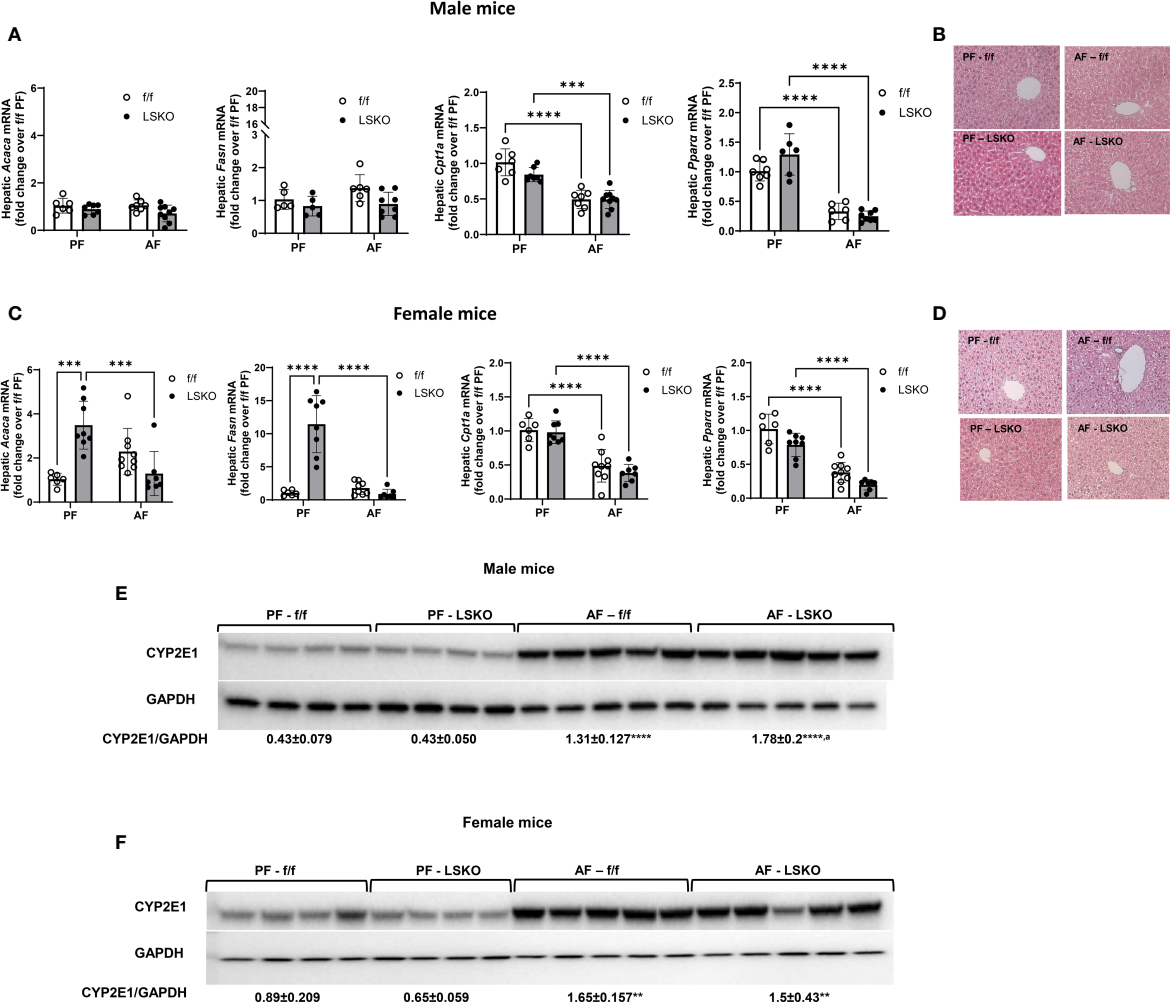
Effect of alcohol and Mkp1 deletion on lipid and ethanol metabolism in males and females. mRNA levels of hepatic acetyl CoA carboxylase 1 (*Acaca*), fatty acid synthase (*Fasn*), carnitine palmitoyltransferase 1A (*Cpt1a*) and peroxisome proliferator activated receptor alpha (*Ppara*) by RT-qPCR analysis in **(A)** males and **(C)** females. Representative images of hematoxylin-eosin staining of liver tissues in **(B)** male and **(D)** female mice. Protein levels of CYP2E1 in **(E)** males and **(F)** females in the chronic plus binge model. Two-way ANOVA **P ≤ 0.01, ***P ≤ 0.001, ****P ≤ 0.0001 compared to their PF counterparts, ^a^P ^≤^ 0.01 compared to AF-f/f.

Steatosis and altered lipid metabolism are hallmarks of ALD. To determine whether Mkp1 deletion influenced pathways involved in alcohol mediated lipid accumulation in the liver, we performed real time qPCR for genes involved in *de novo* lipogenesis, fatty acid synthase (*Fasn*) and acetyl-CoA carboxylase (*Acaca*). Alcohol-fed f/f male mice showed a modest increase in *Fasn* mRNA levels and no change in *Acaca* (**Figure 4A**). In LSKO mice, alcohol feeding had no effect on either *Acaca* or *Fasn*. Carnitine palmitoyl transferase I (*Cpt1a)*, which is essential for fatty acid β-oxidation, was significantly decreased in male f/f and LSKO mice following alcohol feeding (**Figure 4A**). Expression of peroxisome proliferator activated receptor alpha (Pparα), a critical transcription factor for Cpt1a, was also decreased in both genotypes (**Figure 4A**). Baseline mRNA levels of *Acaca* and *Fasn* were significantly higher in PF LSKO females in comparison to f/f (**Figure 4C**), suggesting that Mkp1 deletion increases lipogenesis in female mice. Alcohol feeding led to a modest increase of both *Acaca* and *Fasn* mRNA levels in f/f females but decreased their levels in LSKO females (**Figure 4C**). Both alcohol-fed f/f and LSKO female mice showed decreased levels of *Cpt1 and Pparα* when compared to PF mice. Examination of hematoxylin and eosin-stained slides showed similar lipid accumulation in alcohol fed male mice of both genotypes (**Figure 4B**). In comparison, alcohol fed f/f females had more steatosis when compared to male mice, however LSKO female mice tended to show less steatosis when compared to their alcohol fed f/f counterparts (**Figure 4D**). This observation is consistent with changes in mRNA levels of *Fasn* and *Acaca* (**Figure 4C**). Since CYP2E1-mediated alcohol metabolism plays a major role in alcohol induced liver steatosis and injury, we also examined whether sex and Mkp1 deletion modulate CYP2E1 protein levels. Our data show that CYP2E1 protein expression was significantly increased by alcohol feeding in both f/f males and females (**Figure 4E, F**). However, this increase was significantly higher in LSKO mice when compared to their PF controls. These results suggest that Mkp1 could play a role in ethanol metabolism.

### Effect of sex and Mkp1 deletion on glucose metabolism

**Figure 5:**
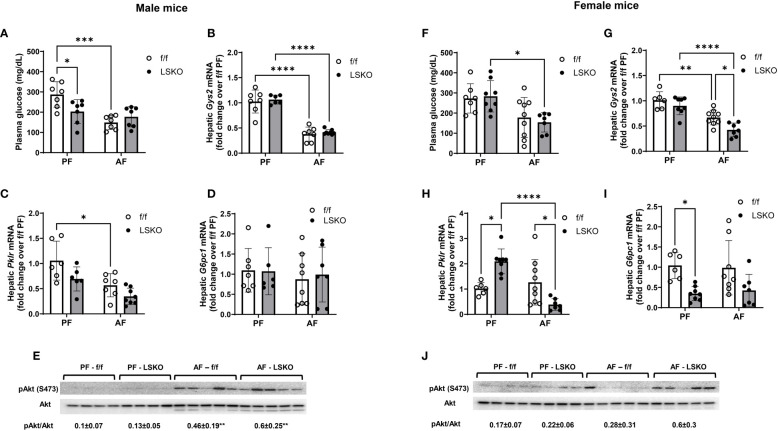
Effect of Mkp1 deletion on hepatic glucose metabolism. **(A)** Plasma glucose levels in male mice, RT-qPCR analysis of hepatic genes in males **(B)** Glycogen synthase (*Gys2*), **(C)** Pyruvate kinase (*Pklr*), **(D)** Glucose-6-phosphatase catalytic subunit 1 (*G6pc1*), **(E)** Western blot analysis of hepatic pAkt and Akt, **(F)** Plasma glucose levels in female mice, RT-qPCR analysis of hepatic genes in females **(G)** Glycogen synthase (*Gys2*), **(H)** Pyruvate kinase (*Pklr*), **(I)** Glucose-6-phosphatase catalytic subunit 1 (*G6pc1*), **(J)** Western blot analysis of hepatic pAkt and Akt. Two-way ANOVA **P ≤ 0.01, ***P ≤ 0.001, ****P ≤ 0.0001.

Recent studies have shown that chronic alcohol consumption is associated with dysregulated glucose metabolism and insulin resistance in the liver (41, 42). Hence, we examined whether Mkp1 deletion influenced glucose metabolism in male and female mice. Baseline plasma glucose levels were lower in PF LSKO male mice when compared to their f/f counterparts (**Figure 5A**). Alcohol feeding decreased glucose levels in male f/f mice but not in LSKO mice (**Figure 5A**). Baseline mRNA levels of Glycogen synthase (*Gys2*), Pyruvate kinase (*Pklr*), and Glucose-6-phosphatase catalytic subunit 1 (*G6pc1*) were not statistically different in f/f and LSKO mice (**Figure 5**). Alcohol did not change mRNA levels of *G6pc1*, but decreased *Gys2* in both f/f and LSKO mice (**Figures 5B, G**)*. Pklr* mRNA levels were significantly decreased only in f/f mice by alcohol feeding (**Figure 5C**). pAkt levels increased in alcohol fed male f/f and LSKO mice, however the levels were slightly higher in AF LSKO mice (**Figure 5E**). In females, alcohol feeding resulted in decreased plasma glucose levels in both f/f and LSKO mice, however in f/f mice it did not reach significance (**Figure 5F**). Baseline levels of *Pklr* mRNA were lower and *G6pc1* higher in LSKO females (**Figures 5G, H**). Alcohol had no effect on the expression of *G6pc1* in either genotype, but decreased *Gys2* levels in both f/f and LSKO females. *Pklr* levels were slightly increased in alcohol fed f/f but decreased in LSKO females (**Figures 5H**). pAkt levels did not increase with alcohol feeding in f/f females, while we saw higher pAkt levels in alcohol fed LSKO females suggesting that Mkp1 deletion influenced insulin sensitivity (**Figure 5J**). Overall, these observations indicate that hepatocyte *Mkp1* deletion influences glucose metabolism and insulin sensitivity in male and female mice differently.

### Effect of Mkp1 deletion on alcohol induced ER stress

**Figure 6:**
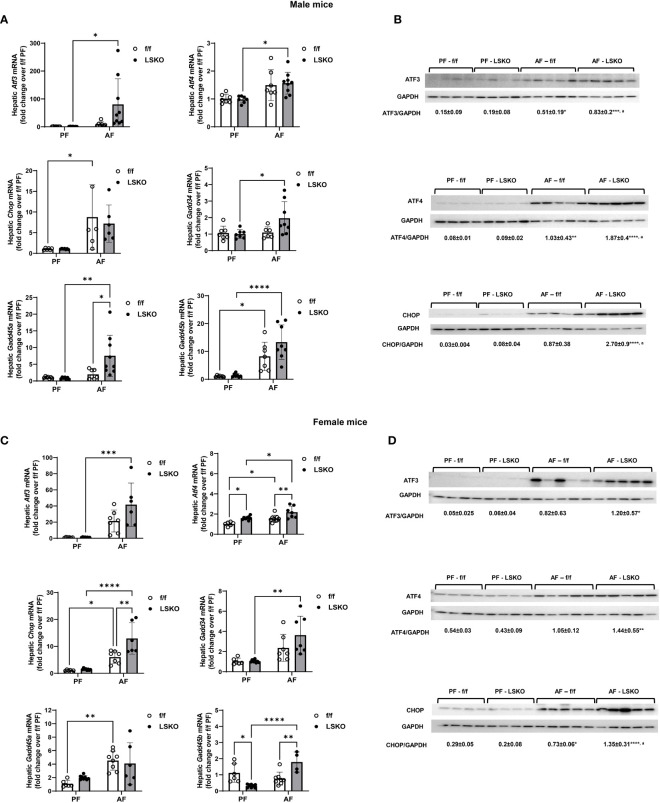
Increased ER stress in alcohol-fed hepatocyte-specific *Mkp1* knockout mice. **(A)** RT-qPCR analysis of genes associated with ER stress in total liver tissue from pair-fed and alcohol-fed male mice in the chronic plus binge model. **(B)** Western blot analysis of hepatic ATF3, ATF4 and CHOP proteins in males. **(C)** hepatic ER stress gene expression in females, **(D)** Western blot analysis of hepatic ATF3, ATF4 and CHOP proteins in females. Two-way ANOVA, *P ≤ 0.05, **P ≤ 0.01, ***P ≤ 0.001, ****P ≤ 0.0001 compared to their PF counterparts, ^a^P ^≤^ 0.01 compared to AF-f/f.

Endoplasmic reticulum stress is strongly associated with ALD and has been shown to contribute to alcohol-induced liver injury (43). To evaluate whether hepatocyte-specific Mkp1 deletion influenced ER stress, we examined the mRNA expression levels of genes associated with ER stress. Real-time qPCR analyses showed that alcohol feeding significantly increased mRNA levels of Chop and *Gadd45b* in f/f male mice. Levels of *Atf3*, *Atf4*, and *Gadd45a* also increased with alcohol exposed f/f males, though the changes did not reach a statistical significance (**Figure 5A**). Notably, Mkp1 deletion resulted in a statistically significant increase in the ER stress response to alcohol as evidenced by a significantly higher mRNA levels of *Atf3*, Atf4, *Gadd34*, *Gadd45a*, and *Gadd45b* in male LSKO mice (**Figure 6A**). In comparison, Mkp1 deletion led to additional increases in mRNA levels of *Atf4, Chop* and *Gadd45b* in alcohol fed LSKO female mice when compared to their f/f counterparts (**Figure 6C**). Western blot analyses of ER stress proteins ATF3, ATF4 and CHOP confirmed that hepatocyte Mkp1 deletion increased the ER stress response in both male and female mice (**Figures 6B, D**).

### Hepatic levels of cytokines and chemokines

**Figure 7:**
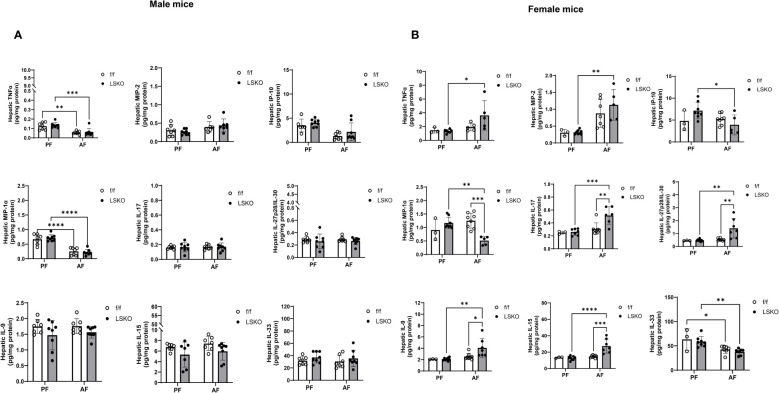
Hepatic levels of cytokines and chemokines. Analysis of cytokines in total liver lysates from **(A)** male and **(B)** female mice subjected to the chronic plus binge model of ALD. Protein levels in total liver lysate were analyzed via MSD Discovery Workbench Desktop Analysis Software. Statistical analysis via Two-way ANOVA, *P ≤ 0.05, **P ≤ 0.01, ***P ≤ 0.001, ****P ≤ 0.0001 compared to their PF counterparts.

Hepatic levels of cytokines and chemokines were analyzed in all treatment groups by using MesoScale Discovery V-PLEX assays. No difference was found between sexes or genotypes in the expression of MCP-1 (Ccl2), IL-1β, IL-4, IL-5, IL-6, IL-10, and IL-12p70 (data not shown). Interestingly, male mice had lower baseline TNFα, IL17 and IL9 protein levels and higher levels of KC-GRO than those from females (**Figure 7**). Alcohol further decreased TNFα levels in males but increased them in female mice. This increase was much more pronounced in LSKO female mice (**Figure 7**). KC-GRO, IL-9, IL-15, IL27p28/IL-30, and IL-17 expression did not change among males of all genotypes and feeding groups. MIP-2, IL-9, IL-15, IL-17, and IL27p28/IL-30 levels were significantly increased with alcohol feeding in LSKO female mice. Expression of IL-33 was unchanged in male mice of all groups but was significantly decreased in female alcohol-fed mice of both genotypes compared to their pair-fed counterparts (**Figure 7**). Overall, we did not observe significant changes in inflammatory response to alcohol in male mice of either genotype in this model. However, there was a modest increase in the response of female mice, which was significantly enhanced by Mkp1 deletion.

## Discussion

Alcohol-associated liver disease (ALD) a common chronic liver disease and is responsible for significant morbidity and mortality worldwide (44). ALD is a general term for a broad spectrum of alcohol-related liver pathologies. ALD pathogenesis begins with simple steatosis (fatty liver) and progresses to steatohepatitis, hepatic fibrosis, and cirrhosis (2, 5). Downregulation of Mkp1 has been observed to be involved in some cases of ALD (24), however, its role has not been fully investigated. The Tsukamoto-French intragastric alcohol feeding model induces a severe liver injury that closely resembles AH pathology in humans. Indeed, our transcriptomic data clearly show that *Dusp1* (gene name for Mkp1) mRNA expression was significantly downregulated in the livers of mice with ASH, similar to what was observed in ALD patients (24). Notably, prior studies by Cederbaum’s group suggested that decreased Mkp1 protein levels are responsible for sustained activation of JNK and CYP2E1-driven liver injury (22).

Our transcriptomic study of livers from mice in ASH study highlighted significant upregulation of most of the dual-specificity phosphatases, specifically dusp 3-6, 8 and 14. These Dusps are well-known as regulators of MAPK pathways, which influence numerous signaling pathways across cell types. They are mostly expressed in immune cells and have been reported to regulate inflammatory responses (30). Dusp1 and its protein form, MKP1, are well-established as crucial regulators of the innate immune response and stimulate the synthesis of numerous proinflammatory factors, including cytokines, chemokines, and other inflammatory mediators (45). MKP1 also regulates the development of an appropriate adaptive immune response: MKP1-deficient innate immune cells also favored CD4+ T cell differentiation toward the Th17 lineage (46). Differential expression of MKP1 is known to influence outcomes in autoimmune diseases (47) and has also been identified as a potential therapeutic target in the management of metabolic disorders, central nervous system disease, cancer, and inflammatory diseases like asthma and sarcoidosis (48). Given the prevalence of MKP1 upregulation in other diseases, its downregulation in ALD hints that its normally protective role is eroded with chronic ethanol exposure. This downregulation aligns with the increased inflammation seen in ALD patients at all stages of disease progression.

After observing that significant downregulation of Mkp1 was associated with increased liver injury and ER stress in the Tsukamoto-French ASH model, we used hepatocyte-specific Mkp1 knockout mice to examine its role in alcohol induced liver injury by using the chronic plusbinge model. This model produces steatosis, liver injury, and ER stress as shown by our group and others (34, 49, 50). Alcohol feeding led a modest increase in liver injury as shown by ALT levels and TUNEL staining (apoptosis index) in f/f male mice, and f/f female mice experienced significant elevation of ALT levels and TUNEL positive cell numbers. We did not observe the same decrease in *Dusp1* mRNA levels in f/f male mice in comparison to ASH mice, possibly due to relatively milder liver injury. However, alcohol had a significant effect of liver *Dusp1* levels in f/f female mice. Notably, alcohol exposure of primary hepatocytes showed that while alcohol decreased Mkp1 mRNA and protein levels in hepatocytes from male mice, alcohol effect was much more pronounced in hepatocytes from female mice. These *in vivo* and *in vitro* observations demonstrate that females are more susceptible to the effects of alcohol exposure on Mkp1 levels. Moreover, these results also suggest that the susceptibility of female mice to alcohol induced injury is at least partially mediated by a differential effect of alcohol on Mkp1 in females. Indeed, we found that hepatocyte-specific deletion of Mkp1 led to increased liver injury in response to alcohol feeding in both female and male mice. This enhanced injury correlated with increased endoplasmic reticulum stress. However, Mkp1 deletion seemed to have more harmful effect on the liver of male mice than in females as demonstrated by higher increase in ALT levels.

Chronic alcohol effects in the liver are largely mediated by the alcohol-metabolizing enzyme, CYP2E1, levels which increase upon alcohol exposure. Our data show that, indeed, CYP2E1 levels increased in alcohol fed female and male mice in both genotypes. However, we did observe that LSKO male mice had a higher increase in CYP2E1 protein levels when compared to their f/f counterparts. We did not observe the same effect of Mkp1 deletion in female mice. This effect of Mkp1 deletion on CYP2E1 levels in male mice could explain the effect we saw on liver injury. Interestingly, Mkp1 deletion had no effect on lipid metabolism in PF or AF male mice. However, baseline levels of lipogenic genes *Acaca* and *Fasn* were significantly higher in female PF mice and were significantly decreased by alcohol feeding. Interestingly, we did not see any effect of Mkp1 on baseline and alcohol-mediated decrease in *Cpt1a* and *Pparα* expression in males and females. Mkp1 deletion also had a sex-dependent effect on baseline glucose levels. Specifically, glucose levels were lower in PF LSKO mice compared to their f/f counterparts. These results are consistent with previous observations that hepatocyte Mkp1 knockout mice have higher levels of insulin (32). Alcohol feeding has been recently shown to increase plasma levels of insulin in chronic plus binge model (51), which explains decreased glucose levels in AF f/f male mice in our study. As for insulin sensitivity, we did not see major changes in males as shown by similar levels of Akt activation. however, in females we did not see the same level of Akt activation in response to alcohol feeding in f/f females which was reflected on glucose levels. Mkp1 deletion seemed to improve insulin sensitivity in females as demonstrated by decreased glucose levels and activation of Akt. This could be due to the differences in baseline insulin levels in f/f and LSKO females, which was not examined in our study. Another intriguing finding of this study was a significantly higher levels of genes involved in lipogenesis (Acaca and Fasn) in LSKO female mice. This could be explained by higher baseline mRNA levels of pyruvate kinase (*Pklr*) in LSKO females. Importantly, *Pklr*, the liver isoform of pyruvate kinase (L-PK or Pklr) is the rate-limiting step in generation of pyruvate and ATP in the cytosol. Mechanistically, Pklr feeds the mitochondria with pyruvate which leads to formation of citrate, a building block for *de novo* lipogenesis through *Acaca* and *Fasn* Of note, recent studies identified Pklr as a novel regulator of liver steatosis (52, 53). Future studies are needed to better understand the mechanisms of sexual dimorphism in Mkp1 regulation of CYP2E1, lipogenesis and glucose metabolism.

The role of Mkp1 in ALD has been examined in a recent study by Li et al. (23). Specifically, the study showed that overexpression of Mkp1 protected mice from alcohol mediated mitochondrial damage, steatosis, inflammation, and injury. Moreover, Mkp1 overexpression prevented alcohol mediated increase in CYP2E1 activity/levels. These results further suggest that Mkp1 has a regulatory role in CYP2E1 expression in male mice. Notably, similar to our results in the ASH model, the authors observed decreased Mkp1 expression in the liver; however, the ALD model and mouse strain they used was different than ours. Specifically, they used the Lieber-deCarli alcohol feeding model for eight weeks in FVB/N mice. Moreover, Mkp1 was overexpressed in the whole body, which is another significant variation from our study. Additionally, they found that Mkp1 overexpression protected mice from alcohol induced downregulation of mitophagy by Mkp1 interaction with Cullin-1 to prevent its nuclear translocation. This aspect of Mkp1-regulation of mitophagy was not examined in our study. However, studies are ongoing to examine this possibility.

Previous studies have examined the dose-response relationship of alcohol and liver injury in females compared to males (54, 55). It is well-established that women are more vulnerable to liver injury with lower volumes of daily alcohol consumption (54, 55); this is reflected in lower daily recommended limits on alcohol consumption for women than men. The reasons behind this dimorphism are widely considered to be multifactorial, and yet, little is known about the organ- and cell-level differences between sexes in ALD. Oxidative stress, endotoxin, and chemokines are known players in the pathogenesis of alcohol-associated liver injury, but few studies have investigated whether they differ between the sexes. In a study using male and female rats, ethanol-fed female rats were found to have more severe liver injury as well as increased endotoxemia, lipid peroxidation, NF-kappa B activation, and upregulation of chemokines MCP-1 (CCL2) and MIP-2 (CXCL2) (56). Similar to our findings, when the NIAAA model of chronic-binge alcohol consumption was used, ethanol-fed female mice had increased ALT levels, MCP-1 mRNA levels, and increased markers of adipose tissue inflammation (57). Notably, these differences in response to ethanol are present even before sex differentiation *in utero* (58 *)*. In *in vitro* studies of mouse embryo cells, female cells were more sensitive to ethanol. Cells from female embryos produced 15% more ROS than male cells in response to ethanol exposure. Though no difference was found between male and female ADH expression, female cells expressed higher levels of CYP2E1 and responded to alcohol with higher increase in CYP2E1 (58). In our studies, both male and female f/f mice had significantly increased hepatic CYP2E1 protein expression with alcohol feeding, as expected. However, the absence of Mkp1 led to increased CYP2E1 protein expression in alcohol-fed males (3 vs 4-fold over PF counterparts). In contrast, alcohol increased the levels similarly in both f/f and LSKO female mice (1.8 vs. 2.3-fold over PF counterparts).

Females have a more robust inflammatory response than males; this is believed to be due to different hormone patterns [estrogens are immune stimulators, while testosterone is somewhat immunosuppressive (59)]. Recent interest in sex differences in ALD has provided a greater understanding of the role of this inflammatory difference in ALD. In one study comparing immunoregulatory cytokines and the impact of Th17 regulatory T-cells in male and female ALD patients, elevated levels of IL-6 and Treg cells were associated with increased severity of liver dysfunction, development of complications, and decreased 90-day survival. Within the study group, females with ALD had significantly greater levels of circulating IL-6 and Treg cells than males, suggesting that an immune difference influences females’ susceptibility to alcohol-associated liver injury (60). In our study, we did not find significant changes in inflammatory cytokines in f/f male and female mice with alcohol feeding; however, Mkp1 deletion increased several cytokine levels in alcohol fed female mice including IL-17. Specifically, cytokines released by macrophages and monocytes (MIP-2, TNFα, IL-9, IL-15, IL-27p28/IL-30) were significantly increased in alcohol fed LSKO female livers. Interestingly, the macrophage-secreted cytokine, IL-33, protein levels decreased with alcohol feeding regardless of Mkp1 expression, which is consistent with a previous report which found that IL-33 levels are decreased in ALD patient livers (61). Further studies are needed to determine whether the increases in proinflammatory mediators seen in female LSKO mice are due to changes in hepatocyte to immune cell crosstalk.

While the chronic plus binge model used in our study is widely used, the exact time point of “peak” inflammation in this model is difficult to track. Because the samples were collected nine hours after binge, data presented are limited to that time point and changes beyond that time point were not captured. Notably, a recent paper using this model did not find any increase in inflammatory cytokines in male mice (51), which is consistent with our previous and recent observations. However, observed elevation in pro-inflammatory cytokines, particularly in female LSKO mice in our study is reassuring. Additionally, the chronic plus binge model successfully recapitulates some, but not all, features observed in human clinical cases of ALD. Future studies are needed to examine the role of hepatocyte specific MKP1 downregulation in ALD progression using other models, particularly those that exhibit more advanced stages of ALD.

## Data availability statement

Liver tissue RNA Sequencing data are available in the NCBI Gene Expression Omnibus, accession number GSE250473. The data can be found here: https://www.ncbi.nlm.nih.gov/geo/query/acc.cgi?acc=GSE250473.

## Ethics statement

The animal study was approved by University of Louisville Institutional Animal Care and Use Committee. The study was conducted in accordance with the local legislation and institutional requirements.

## Author contributions

MW: Data curation, Formal analysis, Investigation, Writing – original draft, Writing – review & editing. DM-D: Data curation, Investigation, Writing – review & editing. WR: Data curation, Investigation, Validation, Writing – review & editing. YW: Data curation, Investigation, Writing – review & editing. JZ: Data curation, Investigation, Writing – review & editing. JC: Data curation, Methodology, Visualization, Writing – review & editing. ER: Methodology, Visualization, Writing – review & editing. CM: Conceptualization, Funding acquisition, Project administration, Writing – review & editing. AB: Conceptualization, Methodology, Resources, Writing – review & editing. CJM: Funding acquisition, Resources, Writing – review & editing. SB: Conceptualization, Methodology, Resources, Supervision, Writing – review & editing. LG: Conceptualization, Funding acquisition, Investigation, Project administration, Supervision, Writing – original draft, Writing – review & editing.
